# HEALTHY AGING AND OLDER ADULTS WITH AUTISM: A SCOPING REVIEW

**DOI:** 10.1093/geroni/igad104.3386

**Published:** 2023-12-21

**Authors:** Mary Stringfellow, Noelle Fields, Kathy Lee, Keith Anderson

**Affiliations:** University of Texas at Arlington, Arlington, Texas, United States; University of Texas at Arlington, Arlington, Texas, United States; University of Texas at Arlington, Arlington, Texas, United States; University of Mississippi, Oxford, Mississippi, United States

## Abstract

Autism Spectrum Disorder (ASD) is a lifelong diagnosis. While several studies have examined the effects of this disorder throughout childhood, few have considered older adulthood and the differences between allistic (i.e., persons who are not affected by autism) and autistic older adults. Moreover, an examination of the intersections of healthy aging in older adulthood and autism are largely absent from extant research. The purpose of this scoping review was to examine the state of literature regarding autistic older adults and the domains of healthy aging, utilizing the framework developed by Arksey and O’Malley (2005). After developing the research question, the researchers followed following the PRISM-ScR guidelines. The specific domains of healthy aging considered included physical health, mobility, mental health, cognition and memory, and social connectedness. A total of 35 articles were selected for final review. There was no definitive age group for autistic older adulthood, therefore the studies’ selection of ages for the older adult population were not homogenous. Fifty-seven percent of these studies considered only one domain of healthy aging, 23% studied two domains, while 14% measured three domains and 6% assessed four domains. No study considered all five domains. Of the five domains considered for this scoping review, mental health and cognition and memory were the most represented domains in the studies. Overall, healthy aging among autistic older adults is largely understudied. More research is needed to better identify and tailor interventions and services for autistic older adults and their families to support healthy aging.

